# Experimental Evaluation of the Effectiveness of Aspiration-Based Techniques to Treat Different Types of Acute Thromboembolic Occlusions in the Femoropopliteal Vascular System Using an In Vitro Flow Model

**DOI:** 10.1007/s00270-021-03024-8

**Published:** 2021-12-17

**Authors:** Schekeb Aludin, Philipp Jost Schäfer, Christoph Borzikowsky, Olav Jansen, Julian Pfarr, Rouven Berndt, René Rusch, Jens Trentmann

**Affiliations:** 1 Department of Radiology and Neuroradiology, University Hospital of Schleswig-Holstein, Kiel, Germany; 2 Institute of Medical Informatics and Statistics, Kiel University, University Hospital Schleswig-Holstein, Kiel, Germany; 3 Department of Cardiovascular Surgery, University Hospital of Schleswig-Holstein, Kiel, Germany

**Keywords:** Aspiration thrombectomy, Thrombectomy, Flow model, Thrombus

## Abstract

**Purpose:**

In this in vitro study, the effectiveness and safety of four aspiration-based techniques for thrombectomy are evaluated for three types of thrombi in a flow model simulating the femoropopliteal segment.

**Material and Methods:**

Red, white, and mixed thrombi were produced in a standardized manner and used to simulate occlusion of a superficial femoral artery using a pulsatile flow model. Four techniques were compared: aspiration alone, aspiration + stent retriever, exposing thrombus to laser by an excimer laser system and a laser catheter + aspiration, and aspiration + mechanical fragmentation by a separator. Rate of first-pass recanalization, embolic events, and number of embolized fragments > 1 mm were compared.

**Results:**

Aspiration alone, stent retriever, laser, and separator differed in rates of first-pass recanalization (53.3%; 86.6%; 20%; and 100%) and embolic events (40%; 93.3%; 73.3%; and 60%). Number of embolized fragments was lowest with aspiration and higher with separator, laser, and stent retriever. Rates of first-pass-recanalization (75%; 75%; and 45%) and embolic events (65%; 60%; and 75%) differed for red, white, and mixed thrombi. The mixed thrombus caused the highest number of embolized fragments, which was particularly high using the stent retriever.

**Conclusion:**

Additional use of mechanical techniques significantly enhances the effectiveness of thrombectomy but simultaneously provokes more embolism. Laser seems to negatively alter the structure of a thrombus and thus diminishes the effectiveness, while provoking embolism. All techniques had lowest effectiveness, but highest embolism with the mixed thrombus. This was particularly striking when a stent retriever was used with the mixed thrombus.

## Introduction

Percutaneous thrombectomy is an important option for endovascular treatment of acute limb ischemia (ALI). One of the oldest techniques here is percutaneous aspiration thrombectomy (PAT), which became the standard endovascular treatment of ALI [1–4]. Subsequently, new endovascular techniques were rapidly developed, e.g., rotational (e.g., Rotarex®; Straub Medical, Switzerland), hydrodynamic (e.g., Angiojet®; Boston Scientific, USA), or mechanical fragmentation thrombectomy (e.g., Separator®; Penumbra, USA) [4–7]. Additionally, techniques for thrombectomy introduced from other vascular territories are also applied: For instance, stent retrievers were originally used to treat acute ischemic stroke (AIS) and intravascular use of excimer laser was mainly applied in cardiointerventional field [8–10]. However, as these techniques prove high effectiveness, they were partly attempted in peripheral vessels, too [11–13]. Nowadays, therefore, interventionalists can choose from among technically heterogeneous devices. Additionally, latest research from neuroradiological field indicates that the type of a thrombotic occlusion seems to influence a device’s performance with thrombectomy [14–16]. Thrombi are heterogeneous and can be classified by pathology, with smooth transitions in between. Red thrombi form quickly under static circumstances and are rich in erythrocytes. They are more likely in venous thrombosis but can also appear in arteries. White thrombi are more common in arterial vessels, form slowly under high shear stress, and precipitate at the vessel’s inner wall. Mixed thrombi partly offer characteristics of both [17, 18]. Several studies examined thrombi extracted from patients with AIS and acute coronary syndrome (ACS), indicating such heterogeneous composition [19–22]. Here, composition seems to influence thrombus mechanics, which may affect a device’s effectiveness [16, 23, 24]. Although data are lacking, this aspect seems to play a relevant role in peripheral thrombectomy, too.

Indeed, it is of major importance to further understand the influence of different techniques for thrombectomy and the varying mechanical features of thrombi on effectiveness and safety. In this in vitro study, therefore, four aspiration-based techniques were evaluated for effectiveness and safety of recanalization in an in vitro flow model providing red, white, and mixed thrombi.

## Material and Methods

### Production of Different Types of Thrombi

Citrated whole blood was acquired from human male volunteers after quality control. The Chandler Loop technique, a common technique for creating dynamic thrombi, was used with a respective device (Acandis, Germany) to produce different types of thrombi [25–27]. Blood was filled into circular PVC tubes with an 8-mm inner diameter and calcium chloride added to each sample for restoring coagulability. The tubes rotated for a period of 90 min at 15 rpm in a tub filled with water at 37 °C.

**Fig. 1:**
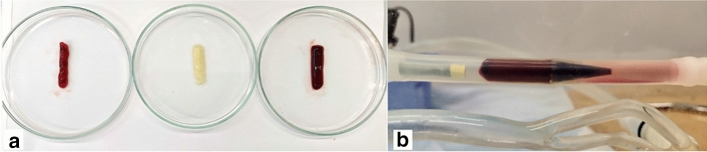
**a** Three different types of thrombi produced in the Chandler loop. Mixed thrombus with white head and red tail (left), white thrombus of pure plasma and rich in fibrin (mid), and red thrombus with homogeneous structure (right). **b** High-grade stenosis segment (ca. 70%) after inserting a representative red thrombus and a guiding catheter proximal to it

A red thrombus was produced by filling the whole tube with blood, yielding thrombi of homogeneous structure and high elasticity (Fig. 1a). A mixed thrombus was produced by only filling half of the tube, resulting in smaller, more fragile thrombi with a typical structure: a small white head, followed by a long red tail (Fig. 1a). A white thrombus was made of pure plasma. Blood was therefore centrifuged at 1800 g for 10 min to separate plasma from corpuscular components. Afterwards, the plasma was isolated, recalcified, and inserted in the Chandler loop, resulting in mostly white thrombi with a very stable and highly elastic structure. As solely plasma was used, this thrombus was assumed to be rich in fibrin and mostly free of erythrocytes (Fig. 1a).

### Flow Model

**Fig. 2:**
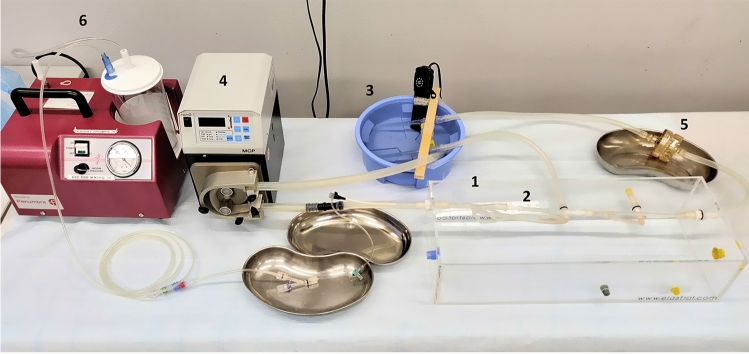
Experimental setup, consisting of vessel phantom (1) with stenosis segment (2), reservoir of saline (3), pulsatile pump (4), distal filter system (5), and vacuum pump (6)

A circular system of silicon tubes connected to a vessel phantom formed the flow model (Elastrat Sarl, Switzerland). The vessel phantom consisted of a glass basin with fixed silicon tubes (Fig. 2), which resembled the vascular structure of a common femoral artery, followed by the bifurcation into a superficial femoral artery (SFA) and a deep femoral artery. The SFA had a removable segment with an inner diameter of 5 mm, in which a high-grade stenosis of ca. 70% was located. The vessel phantom was integrated in a circular system, consisting of a reservoir filled with saline at 37 °C and a pulsatile pump. Furthermore, a filter system for examining peripheral embolism was located distal from the stenosis. For inserting the thrombi into the removable segment, the standardized length of each thrombus was set at 35 mm. Larger thrombi were cut to fitting length. The thrombi caused a standardized occlusion, and by running the pump at 70 bpm, the mean intravascular pressure was 85 mmHg (Fig. 1b).

### Devices and Procedures for Thrombectomy

Four thrombectomy devices were examined: (1) aspiration alone by using a guiding catheter (GC) (Mach 1 peripheral 7F; Boston scientific, USA); (2) aspiration with additional use of a stent retriever (Trevo XP Pro Vue 6 × 25 mm; Stryker, USA); (3) exposing the thrombus to laser from an excimer laser system (CVX-300; Spectranetics, USA) and via a laser catheter (TurboElite 1.4 mm OTW; Spectranetics, USA) prior to aspiration; and (4) aspiration with additional mechanical clot fragmentation by using a separator (Separator; Penumbra, USA).

The experiment starts with placing a thrombus in the stenotic segment. Afterward, a guidewire (Synchro2; Stryker, USA) was introduced to the flow model prior to thrombectomy. For each maneuver, standardized aspiration with a vacuum pump (Penumbra, USA) connected to the GC was employed. No system for flow arrest was utilized.

#### Aspiration

PAT was performed by pushing the GC into the proximal part of the occluding thrombotic material. After placement, aspiration was started by turning on the vacuum pump. The GC was left in position for approximately 5 s to evacuate thrombotic material. Subsequently, the GC was slowly drawn back in retrograde direction. Aspiration was permanently maintained during withdrawal until the GC was pulled out through the arterial introduction sheath.

#### Aspiration + Stent Retriever

A microcatheter (Trevo Pro18; Stryker, USA) was placed beyond the stenosis. By pulling it back, the stent struts expanded on the thrombus and the stent was set to rest for three minutes for full expansion. Afterward, aspiration was started and the stent retriever-thrombus unit slowly drawn back into the GC. If the GC happened to occlude and the vacuum pump detected flow arrest, the whole unit was drawn back through the arterial introduction sheath.

#### Laser Exposure Prior to Aspiration

The laser catheter was introduced and the tip placed directly proximal to the thrombus. The excimer laser system was activated and the laser catheter pushed through the thrombus once in an antegrade and once in a retrograde direction at a speed of 1 mm/s. Additionally, its tip was simultaneously flushed with saline. After passage, the laser catheter was drawn back through the arterial introduction sheath and aspiration maneuvers were performed with the GC, as described above in section “*Aspiration*”.

#### Aspiration + Mechanical Fragmentation by a Separator

The separator consists of a wire with a plastic olive on the tip. The GC was placed proximal to the thrombus and the separator pushed through the GC until the olive was 1 cm distal to the GC’s tip and directly proximal to the thrombus. Aspiration was initiated and the GC-separator unit pushed through the thrombus to evacuate thrombotic material. If the GC became occluded, the separator was drawn back and the olive was inserted into the tip of the GC. This movement was performed until the occluding thrombotic material was fragmented and could be aspirated.

If the GC became occluded with thrombotic material, it was drawn back through the arterial introduction sheath, as described above. As assistance here, single additional manual aspiration was performed through a 20-ml syringe (a 20-ml syringe was used instead of a syringe with higher volume, as a single person could control it better). The devices were used for each type of thrombus in 5 experiments, yielding 15 experiments per device and 60 experiments in total (*n* = 60). Recanalization after first maneuver was defined as first-pass recanalization rate (FPR). If the occlusion persisted after this first thrombectomy maneuver, the FPR was noted as negative and up to two further thrombectomy maneuvers could be performed to recanalize the vessel. All three maneuvers for thrombectomy were counted for one single experiment. After each experiment, the distal filter system was examined visually with a magnifier to detect embolized fragments, which were measured with a precision caliper. If embolized fragments larger than 1 mm were present, this was documented positively as embolic event (EE). Additionally, embolized fragments larger than 1 mm were also counted and documented as number of embolized fragments (NEF).

### Statistics

The data are summarized as descriptive statistics, with median (MD), interquartile range (IQR), minimum (Min), and maximum values (Max). The data are visualized as bar diagrams and box plots. Statistical testing was performed by using the Kruskal–Wallis test, which is a nonparametric test for comparisons between more than two groups. Additionally, Chi-squared-test and Fisher’s-exact-test were applied. The statistical significance level was α = 5%. The Bonferroni method was used to correct for multiple comparisons.

## Results

### Rate of First-Pass Recanalization

**Fig. 3:**
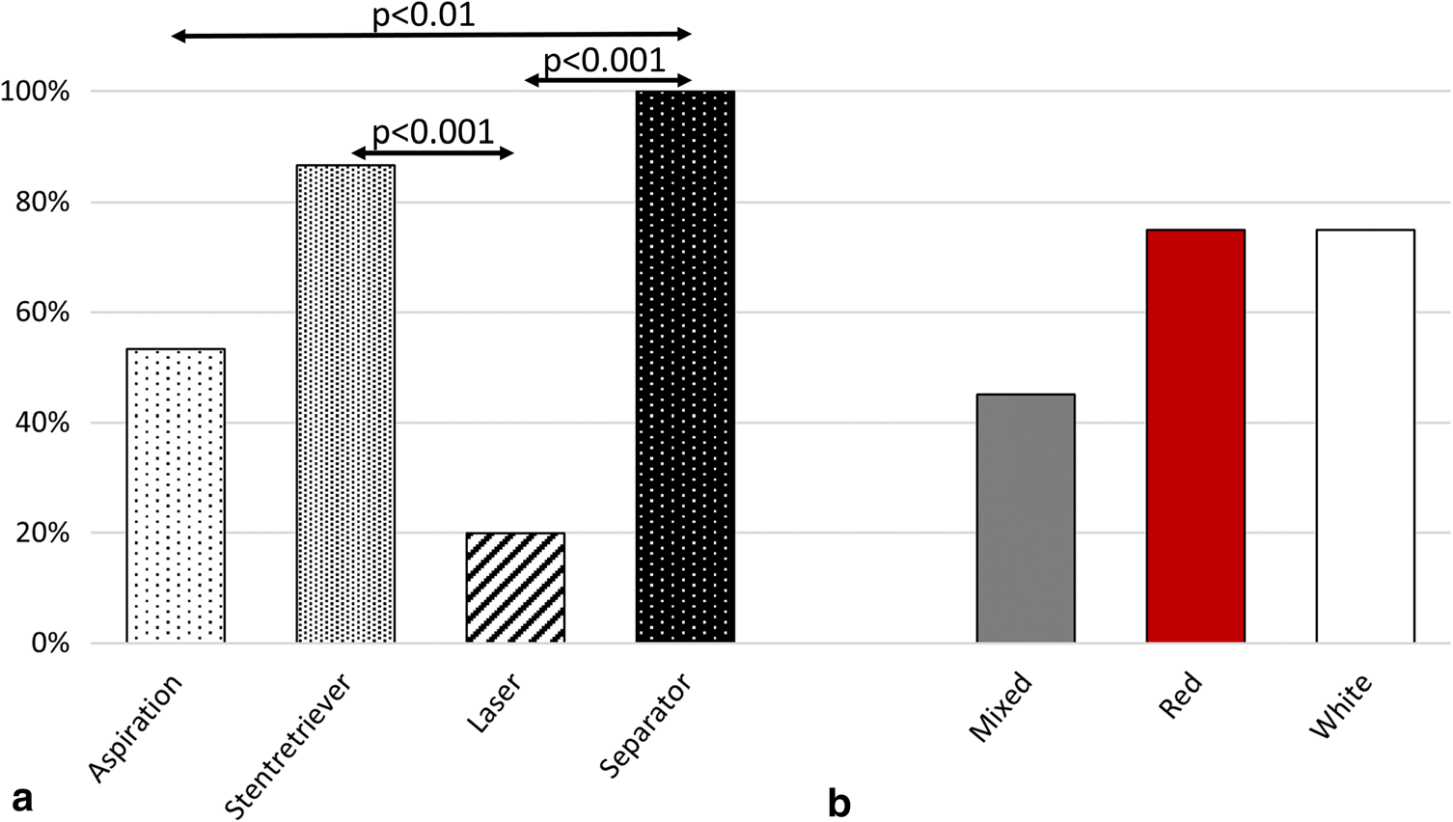
**a** First-pass rate (FPR) for each device (%), regardless of the type of thrombus. **b** FPR (%) with each type of thrombus, regardless of the device

The FPR among all experimental attempts was 39/60 (65%). Regarding each device, the FPR was relatively higher when additionally using the mechanical devices stent retriever (86.6%) and separator (100%) than it was with aspiration alone (53.3%). FPR of the separator was significantly higher than that of aspiration alone (*p* < 0.01). In contrast, exposing the thrombus to laser (20%) seemed more to diminish FPR relative to aspiration alone. FPR of the separator and stent retriever was significantly higher than that of laser (*p* < 0.001) (Fig. 3a). Regarding the different types of thrombi, the mixed thrombus was associated with the lowest FPR (45%), whereas white (75%) and red (75%) thrombi were equally high (Fig. 3b).

### Embolic Events

**Fig. 4:**
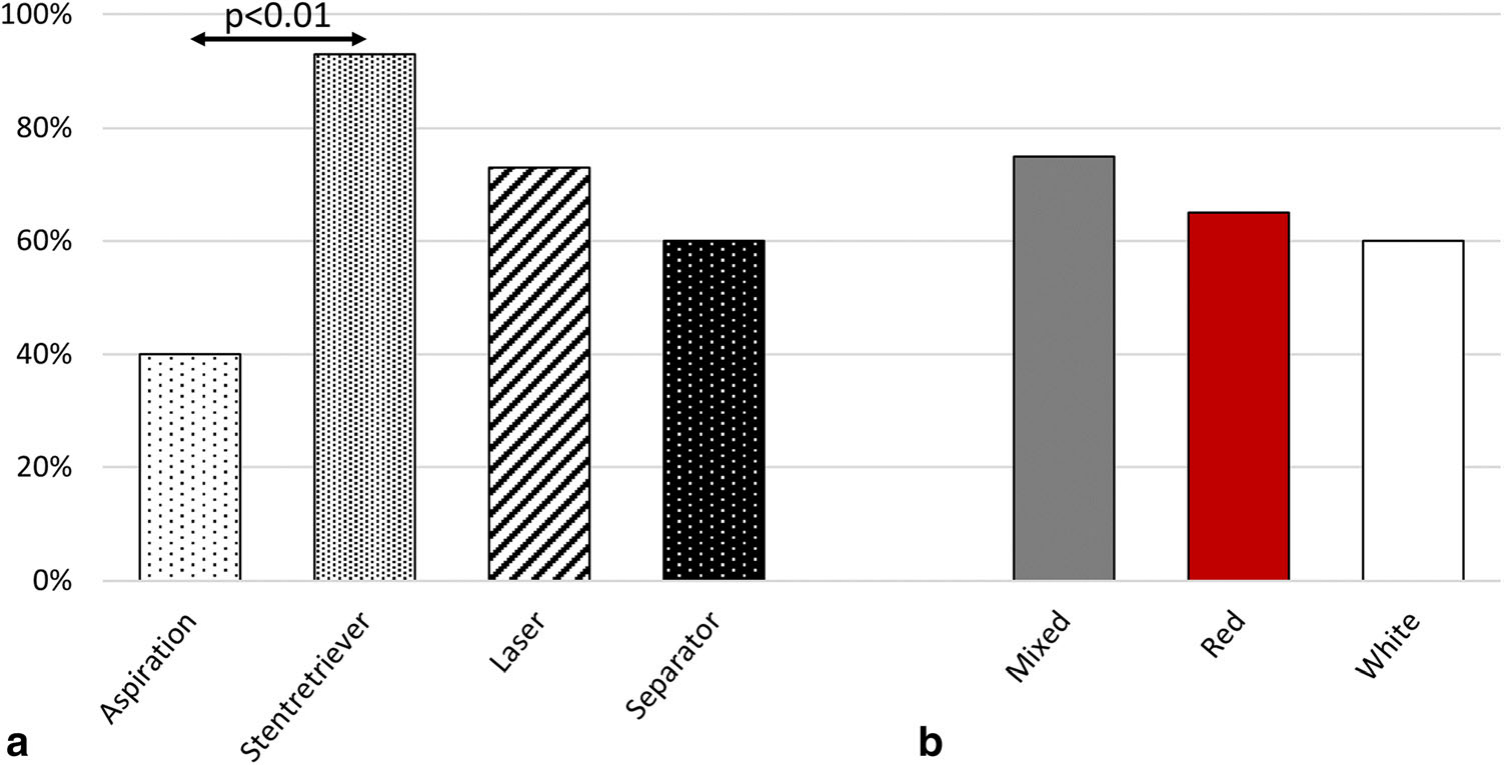
**a** Embolic events (EE) for each device (%), regardless of the type of thrombus. **b** EE (%) with each type of thrombus, regardless of the device

Embolic events occurred in 40/60 (66.6%) of all experimental attempts, the rate thus being lower for aspiration alone (40%) than for the other methods. The stent retriever (93.3%) caused EE in nearly all experimental attempts, followed by laser (73.3%) and separator (60%). EE of the stent retriever was significantly higher than that of aspiration alone (*p* < 0.01) (Fig. 4a). Among the different types of thrombi, the highest EE was associated with the mixed thrombus (75%) and was lower for red (65%) and white thrombi (60%) (Fig. 4b).

### Number of Embolized Fragments

**Fig. 5:**
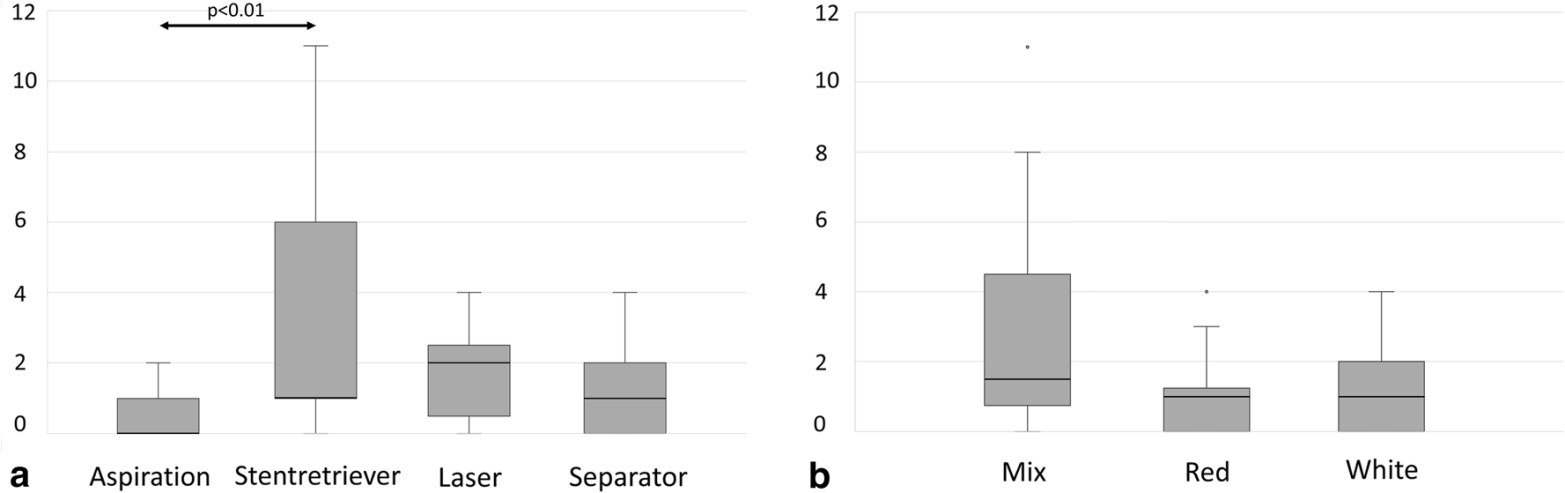
Number of embolized fragments (NEF). **a** With each device. **b** With each type of thrombus

**Fig. 6:**
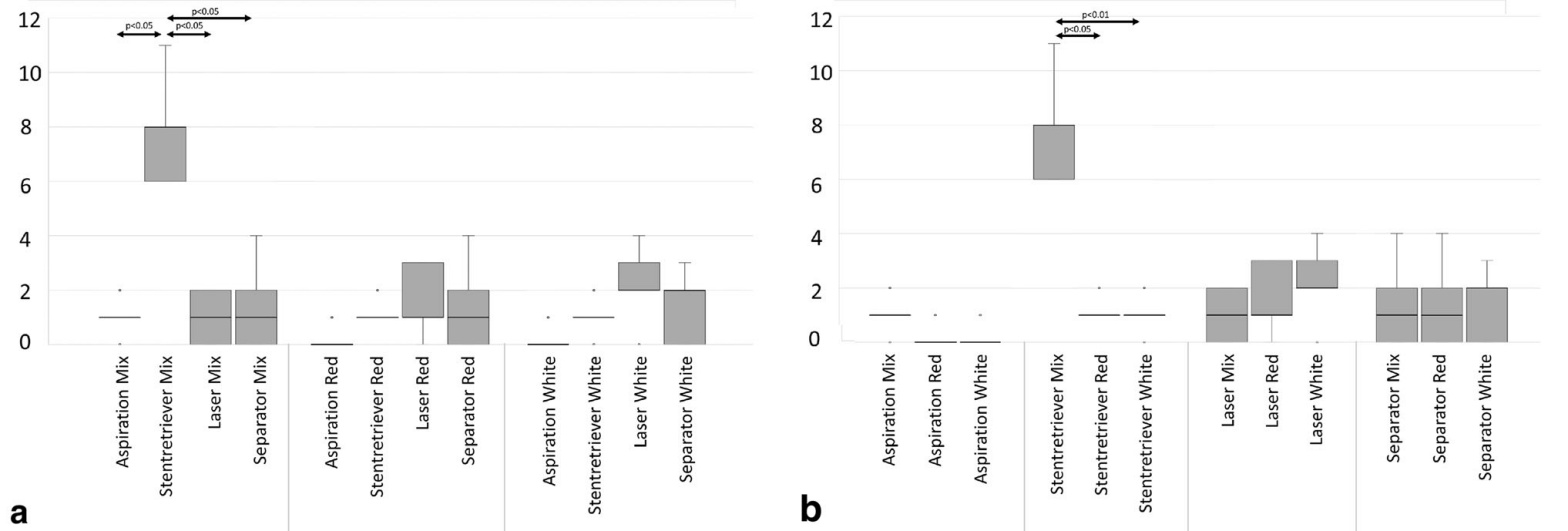
Number of embolized fragments (NEF). **a** With each type of thrombus and with respect to each device. **b** With each device and with respect to each type of thrombus

The NEF was lowest with aspiration alone and, in contrast, highest with the stent retriever, followed by laser and separator. Here, the stent retriever produced significantly more NEF than aspiration alone (*p* < 0.01) (Fig. 5a). Regarding the different thrombi and their potential for causing NEF, the mixed thrombus showed higher NEF than the red and white thrombi (Fig. 5b). The NEF was particularly high when the stent retriever was combined with the mixed thrombus. This combination caused significantly higher NEF than any other device with the mixed thrombus (*p* < 0.05) (Fig. 6a). Furthermore, NEF was also significantly higher than when combining the stent retriever with the red (*p* < 0.05) and white thrombi (*p* < 0.01) (Fig. 6b).

## Discussion

In this in vitro study, an acute thrombotic occlusion of the superficial femoral artery was simulated by three different types of artificial thrombi. Effectiveness and safety of four aspiration-based thrombectomy techniques were evaluated, determining FPR, EE, and NEF.

FPR was higher when aspiration was combined with mechanical devices than when used alone. Hereby, the stent retriever effectively fixed the thrombus during evacuation and the separator cleared the occluding thrombus mass from the catheter tip. However, regarding distal embolism, EE and NEF were higher when other devices were additionally used. Other in vitro studies have investigated devices for peripheral thrombectomy: Muller-Hulsbeck et al. investigated the effectiveness of PAT with red thrombi in an in vitro model of the femoropopliteal segment [28]. Recanalization was achieved in all cases. In our study, the FPR of PAT with the red thrombus was slightly lower at 80%. However, prior investigators did not determine the FPR but only the overall recanalization rate after an unknown number of thrombectomy maneuvers. Accordingly, the FPR may have been lower [28]. Rusch et al. also investigated PAT using an in vitro model with three thrombus entities, like in our study. In that study, the FPR for PAT was 91.6% among all experiments, compared to 53.3% in ours. Reasons for this discrepancy could be the different experimental design. For example, in our study a stenosis of ca. 70% was used, compared to < 50% in prior study. Furthermore, those investigators focused on developing a physiological circulation model. Their thrombectomy procedures were explicitly considered pilot experiments, while ours focused on standardized conditions for performing thrombectomy [29]. Such effects underline the importance of precisely planning and describing the experimental design and the difficulty of comparing different in vitro studies.

In vitro studies of stent retrievers and separators in peripheral vessels have not been conducted, but they have been investigated in other vascular models [30–32]. However, comparisons with these studies should only be made carefully, due to the greatly differing experimental designs. Nevertheless, there is consensus that these devices demonstrate high effectiveness, as in the current study [30–32]. Another important aspect of stent retrievers is their relatively high EE. The technical reasons are not entirely clear, but vascular geometry might explain this. Intracranial vessels are significantly smaller and more tortuous, which may positively affect flow behavior, radial force, and performance during the retrieval maneuver [32, 33]. Furthermore, no systems for flow arrest were used in our study, which are more often used in intracranial thrombectomy and can effectively reduce embolism [34, 35]. Regarding the small diameter of cerebral vessels, it may also be assumed that the aspiration catheter’s diameter itself already causes a certain obliteration of the vessel ‘s lumen and thus lower risk of embolism.

Most other in vitro studies on peripheral thrombectomy are difficult to compare with the current experimental setting, as different techniques like rotational or hydrodynamic thrombectomy are used. However, despite different techniques, these studies do also implicate a certain positive effect of mechanical systems on thrombectomy, consistent with this study [5, 6, 36].

Excimer laser is occasionally used for cardiointerventional recanalization, while studies in peripheral vessels are sparse and limited to chronic occlusions. However, in these studies, prior use of a laser partly increases the effectiveness of subsequent percutaneous transluminal angioplasty [9–12]. In fact, excimer laser can ablate plaque and alter thrombotic material through photothermal and photochemical effects, which may affect thrombectomy [9, 37, 38]. In our study, laser exposure seemed to induce structural alterations in all three types of thrombi, which also made them more fragile. This resulted in a lower FPR, but higher EE and NEF in our model of ALI. Of course, these in vitro results do not necessarily implicate a negative effect in clinical use. Yet, they underscore the danger of thrombus disruption and importance of maintaining a certain mechanical integrity of the thrombus during thrombectomy.

The importance of thrombus structure is further demonstrated in our study. While the red and white thrombus were relatively stable, the mixed thrombus was the most fragile and had the highest tendency for embolization. Different from AIS and ACS, thrombus composition and its influence have not received much clinical attention in ALI yet and studies are not available [14, 15, 22]. However, our results suggest its significance for peripheral thrombectomy and potential clinical relevance. In fact, by using newly developed imaging techniques in computed tomography, magnetic resonance tomography, and ultrasound, tissues can be characterized beyond the realm of neuroradiological applications [39–41]. Peripheral use of such could significantly contribute to the success of peripheral thrombectomy regarding thrombus heterogeneity.

This study has limitations, and the most striking one is its experimental character. Despite the pressure conditions within the model and the heterogeneity of the manufactured thrombi, the study lacks certain in vivo features (e.g., atherosclerotic vessel wall, hemodynamic variances during intervention, and interaction between thrombi and vessel wall). However, the results among the different devices and types of thrombi acquired in this experimental study can contribute to and encourage further research aiming for an even more realistic set up and thus precise testing.

## Conclusion

The study presents an overview of the dynamic process of thrombectomy with different aspiration-based techniques and their interaction with different types of thrombi. The study’s key results are:

(I) The additional use of mechanical devices increases the effectiveness of thrombectomy compared to aspiration alone.
(II) Mechanical manipulation of a thrombus, which is inherent to the use of mechanical devices, also provokes significantly higher NEF. This applies to the strong mechanical manipulation caused by a stent retriever and prior degradation of a thrombus with a laser catheter.
(III) The entity of a thrombus and its associated composition and mechanical features have an impact on the effectiveness and safety of a device. Particularly, mixed thrombi are more fragile and thus associated with lower effectiveness and a higher risk of embolism. These effects are especially confirmed when a stent retriever is used for this type of thrombus.
